# Supplementary material for: Proteomic subtyping of Alzheimer's disease CSF links blood–brain barrier dysfunction to reduced levels of tau and synaptic biomarkers
Source: Alzheimers Dement. 2025 Nov 3;21(11):e70830. doi: 10.1002/alz.70830 (PMC12580855; doi:10.1002/alz.70830)
Supplement: Supplementary file 1 — Supporting Information [file ALZ-21-e70830-s004.pdf]

Supplementary Figure 1

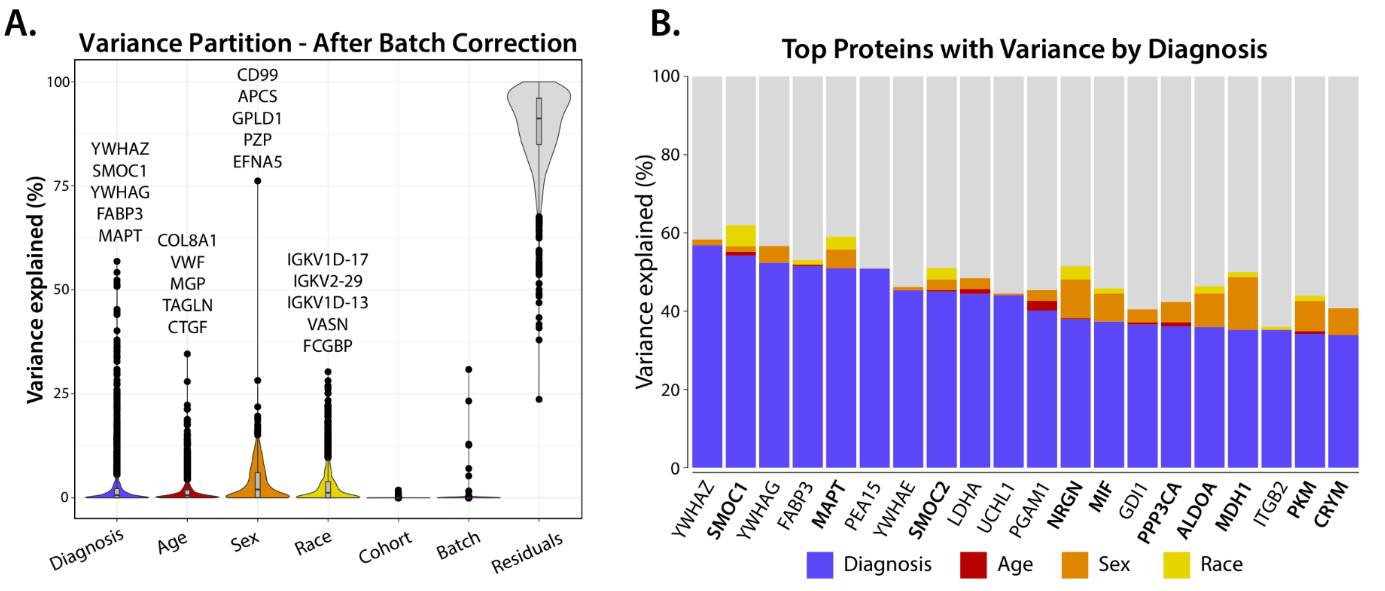

Supplemental Figure 1: Variance partition analysis reveals that proteins associated with AD diagnosis differ based on race and sex. (A) Variance partition analysis was performed to determine to what magnitude the factors of diagnosis, age, race, and sex contributed to variations in cohort protein abundance, and to confirm minimal contributions to data variance due to cohort or TMT batch. The top 5 proteins with the greatest variance in abundance attributable to each factor are noted. **(B)** The top 20 proteins with the greatest variance in abundance across cases due to AD diagnosis also have notable contributions to their variance attributable to the sex, race, and age of the participant. Bolded proteins labels indicate proteins where the combined variance due to race and sex is greater than 10% of that due to diagnosis.
