# Supplementary material for: Proteomic subtyping of Alzheimer's disease CSF links blood–brain barrier dysfunction to reduced levels of tau and synaptic biomarkers
Source: Alzheimers Dement. 2025 Nov 3;21(11):e70830. doi: 10.1002/alz.70830 (PMC12580855; doi:10.1002/alz.70830)
Supplement: Supplementary file 2 — Supporting Information [file ALZ-21-e70830-s010.pdf]

Supplementary Figure 2

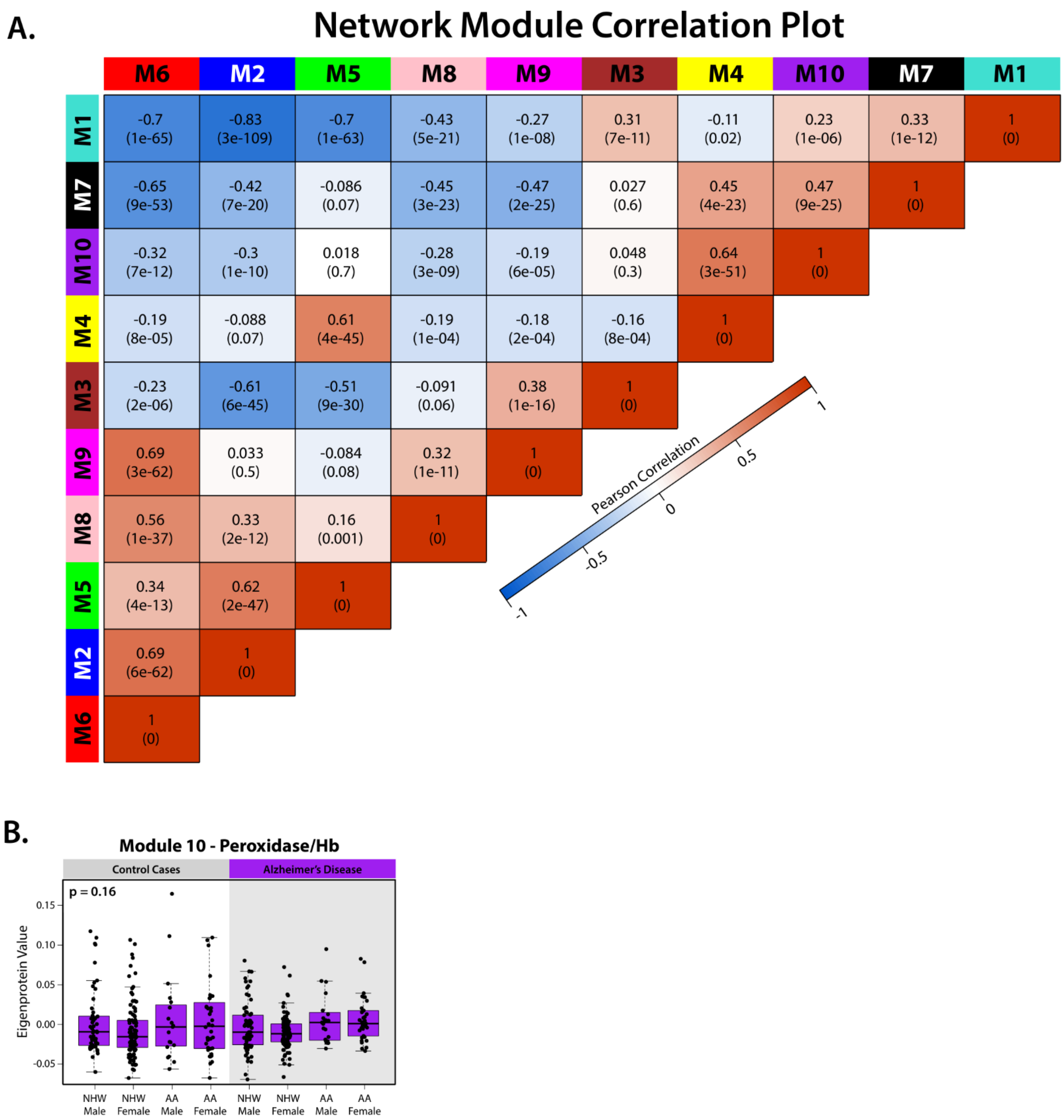

Supplemental Figure 2: Module correlations within the co-expression network. (A) Pearson correlations and p values corresponding to protein modules within the network demonstrating positive (red) or negative (blue) associations. **(B)** Eigenprotein values from Module 10 broken out by participant demographic. Significance assessed by 1-way ANOVA.
