# Supplementary material for: Proteomic subtyping of Alzheimer's disease CSF links blood–brain barrier dysfunction to reduced levels of tau and synaptic biomarkers
Source: Alzheimers Dement. 2025 Nov 3;21(11):e70830. doi: 10.1002/alz.70830 (PMC12580855; doi:10.1002/alz.70830)
Supplement: Supplementary file 3 — Supporting Information [file ALZ-21-e70830-s001.pdf]

Supplementary Figure 3

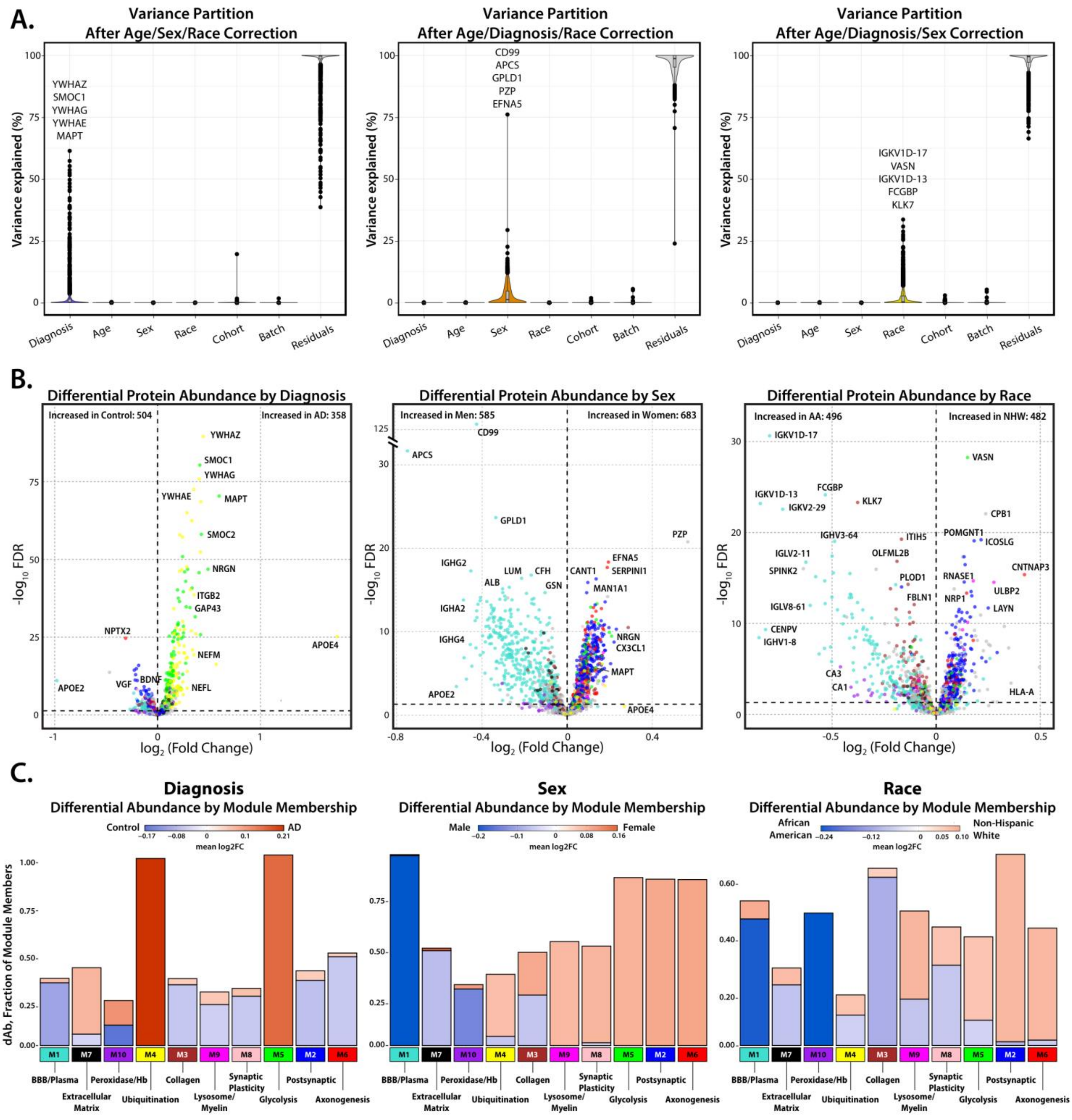

Supplemental Figure 3: Influence of Diagnosis, Sex, and Race on Protein Variance and Module Abundance in the Emory Cohort. **(A)** Variance partition analysis of the Emory cohort regressed for all variables except diagnosis (**left**), sex (**center**) and race (**right**) demonstrate the effective minimization of other sources of variance. Plots are annotated with the top 5 proteins with the greatest variance in abundance due to each factor. **(B)** Volcano plots displaying the log2-fold change of protein abundance plotted against the FDR corrected 1-Way ANOVA p value, demonstrate the differential protein abundance of the preserved factors of diagnosis (**left**), sex (**center**) and race (**right**) in the absence of other variables. Proteins are annotated by module color, illustrating the relationships between modules and demographic factors. **(C)** A fractional breakdown of the proteins in each module that have significant differential abundance based on the selected factors of diagnosis (**left,** blue: control, red: AD), sex (**center,** blue: male, red: female) and race (**right,** blue: AA, red: NHW), highlighting the fundamental influence of diagnosis and demographics on module abundance, even in the absence of the other sources of variance.
