# Supplementary material for: Proteomic subtyping of Alzheimer's disease CSF links blood–brain barrier dysfunction to reduced levels of tau and synaptic biomarkers
Source: Alzheimers Dement. 2025 Nov 3;21(11):e70830. doi: 10.1002/alz.70830 (PMC12580855; doi:10.1002/alz.70830)
Supplement: Supplementary file 4 — Supporting Information [file ALZ-21-e70830-s003.pdf]

Supplementary Figure 4

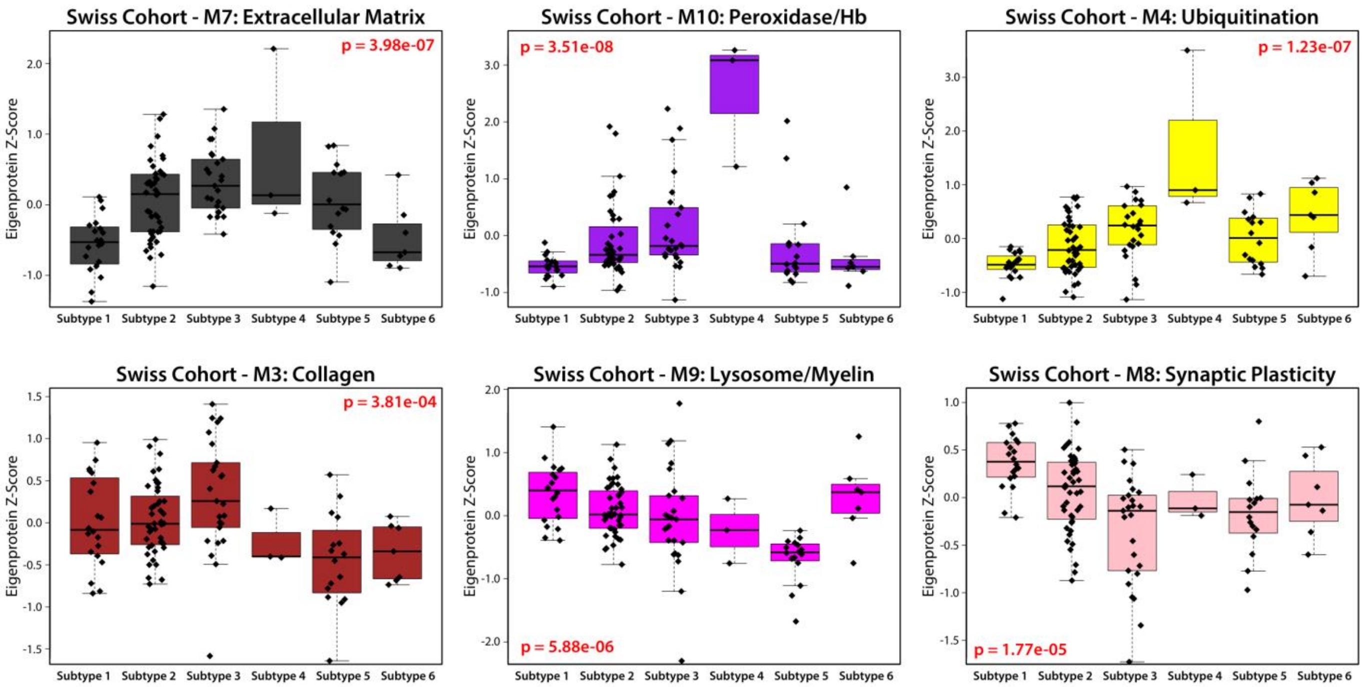

Supplemental Figure 4: Network Module Eigenprotein Levels Across UMAP Subtypes in the Swiss Replication Cohort. Additional network module eigenprotein boxplots for the Swiss Replication cohort, broken out by assigned UMAP subtype in order of module relatedness. Significance was assessed by 1-way ANOVA.
