# Supplementary material for: Proteomic subtyping of Alzheimer's disease CSF links blood–brain barrier dysfunction to reduced levels of tau and synaptic biomarkers
Source: Alzheimers Dement. 2025 Nov 3;21(11):e70830. doi: 10.1002/alz.70830 (PMC12580855; doi:10.1002/alz.70830)
Supplement: Supplementary file 5 — Supporting Information [file ALZ-21-e70830-s006.pdf]

Supplementary Figure 5

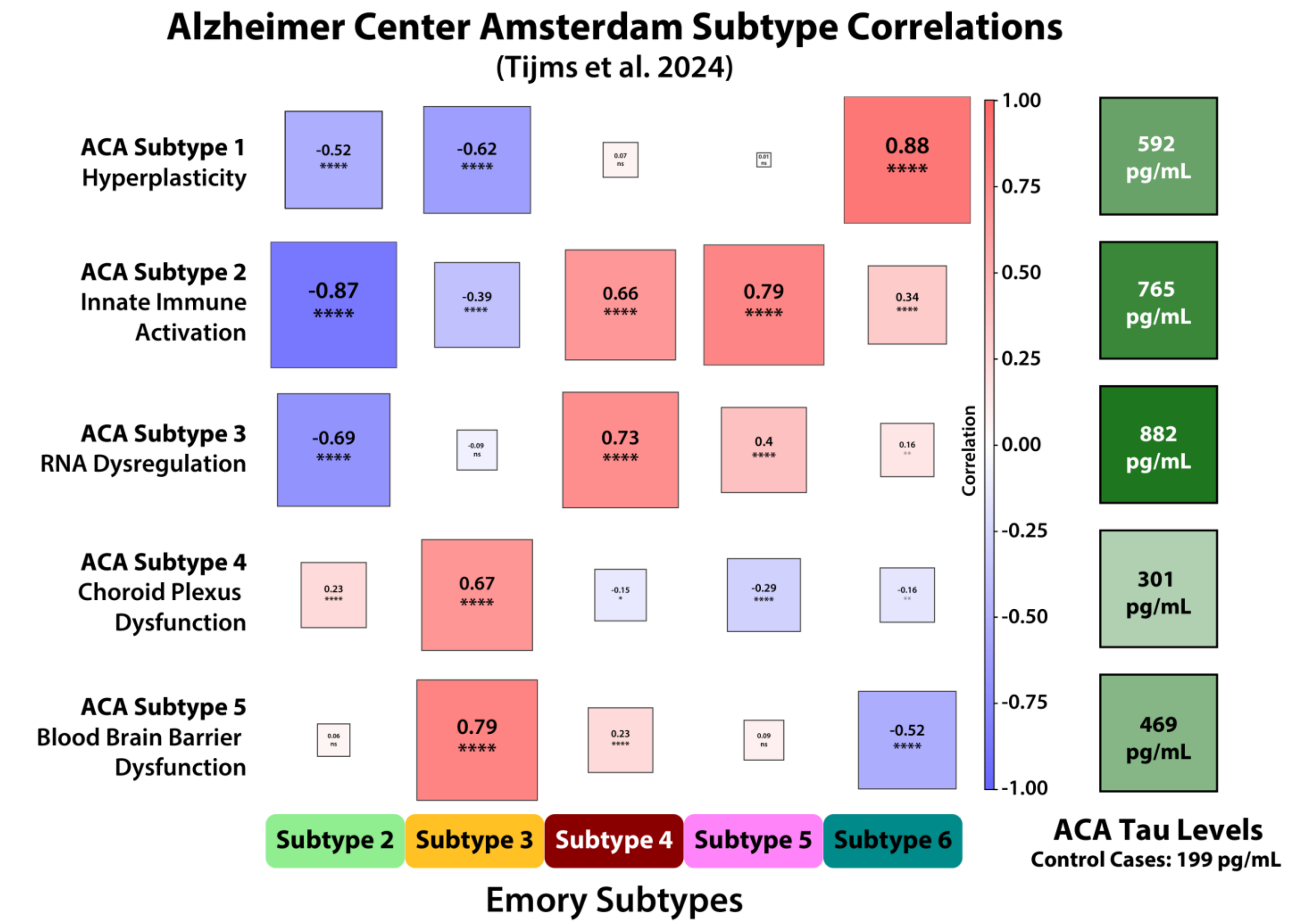

Supplemental Figure 5: Correlation between the Emory and Alzheimer's Center Amsterdam Subtypes. (A) A heatmap showing the correlation between the z-scored mean protein abundances of participants in the Emory and Alzheimer's Center Amsterdam (ACA) proteomic subtypes. Subtypes with strong positive (red) or negative (blue) bicor values between the cohorts indicate relatedness (*p≤0.05; **p≤0.01; ***p≤0.001 ****p≤0.0001). **(B)** Corresponding immunoassay CSF Tau levels from each ACA subtype, where all participants have been diagnosed with AD. ACA subtypes that were highly correlated with Emory Subtype 3 (ACA Subtype 4/Choroid Plexus Dysfunction, ACA Subtype 5/Blood Brain Barrier Dysfunction) also had the lowest levels of CSF Tau.
