# Supplementary figures and images for: Proteomic subtyping of Alzheimer's disease CSF links blood–brain barrier dysfunction to reduced levels of tau and synaptic biomarkers
Source: Alzheimers Dement. 2025 Nov 3;21(11):e70830. doi: 10.1002/alz.70830 (PMC12580855; doi:10.1002/alz.70830)

# Supplementary Figure 6

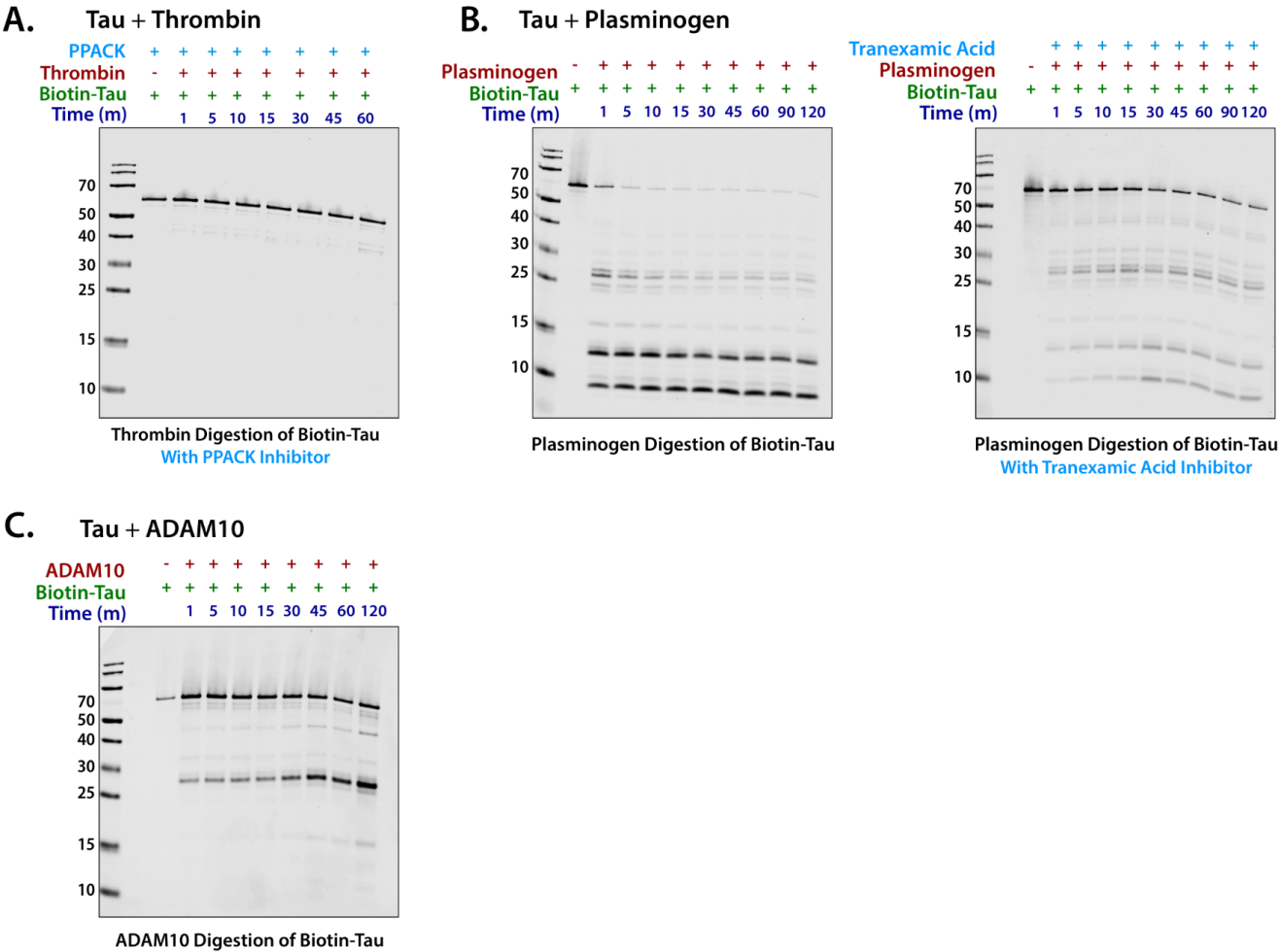

Supplement: Supplementary file 6 — Supporting Information [file ALZ-21-e70830-s009.pdf]
