# Supplementary material for: Proteomic subtyping of Alzheimer's disease CSF links blood–brain barrier dysfunction to reduced levels of tau and synaptic biomarkers
Source: Alzheimers Dement. 2025 Nov 3;21(11):e70830. doi: 10.1002/alz.70830 (PMC12580855; doi:10.1002/alz.70830)
Supplement: Supplementary file 7 — Supporting Information [file ALZ-21-e70830-s002.pdf]

Supplementary Figure 7

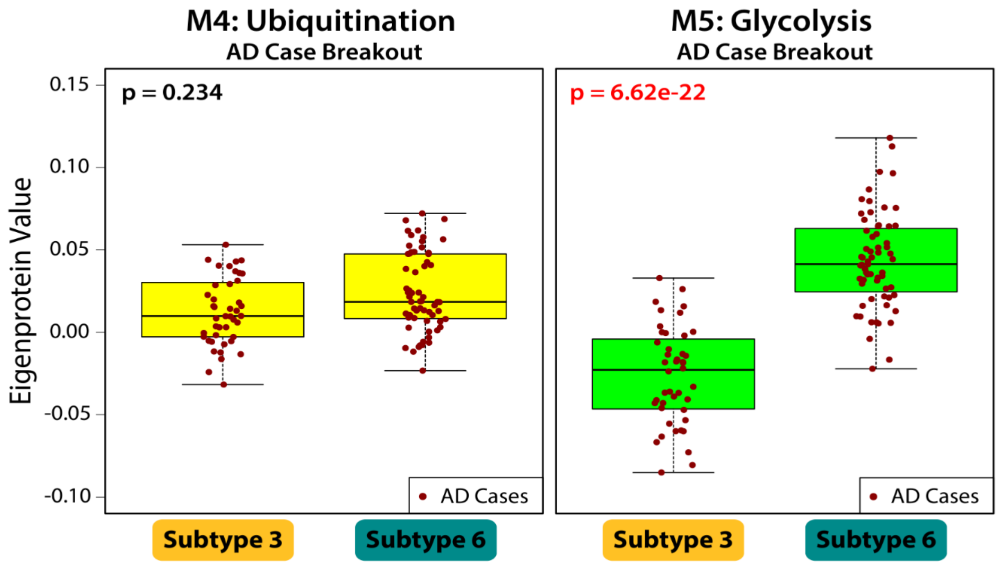

Supplemental Figure 7: Comparison of AD Pathology-Associated CSF Modules Between Subtype 6 and Subtype 3 AD Samples. A breakout of exclusively the AD cases from Subtype 6 and Subtype 3, comparing levels of the two AD pathology associated modules, M5: Glycolysis, and M4: Ubiquitination. While there is a significant difference in M5 levels in AD cases between the two subtypes, there is no significant difference in M4 levels (as assessed by 1-way ANOVA).
