# Supplementary material for: Proteomic subtyping of Alzheimer's disease CSF links blood–brain barrier dysfunction to reduced levels of tau and synaptic biomarkers
Source: Alzheimers Dement. 2025 Nov 3;21(11):e70830. doi: 10.1002/alz.70830 (PMC12580855; doi:10.1002/alz.70830)
Supplement: Supplementary file 8 — Supporting Information [file ALZ-21-e70830-s008.pdf]

Supplementary Figure 8

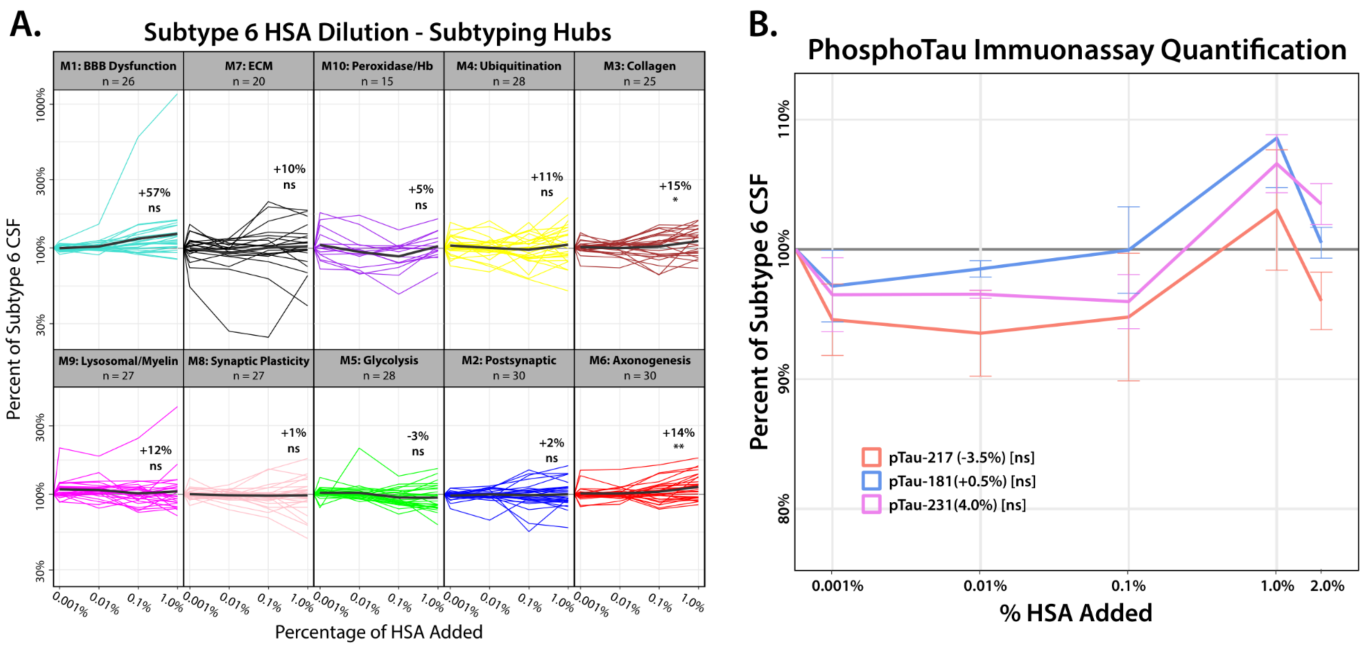

Supplemental Figure 8: Impact of Human Serum Albumin (HSA) Dilution on Network Module Proteins and Phosphorylated Tau Levels in AD-like Subtype 6 CSF. (A) DIA-MS analysis of HSA doping was run in parallel to plasma doping experiments. Proteins that overlapped with the top 30 network hub proteins used to subtype the Emory Cohort were broken out by module, and plotted across increasing amounts of HSA, normalized as a percentage of their abundance in pooled Subtype 6. Bold black lines represent the average percent change across the subtyping hub proteins for each module. Significance was determined by Tukey adjusted 1-way related measures ANOVA comparing initial and final concentrations of proteins within each module. **(B)** Levels of endogenous phosphorylated tau (pTau$_{181}$, pTau$_{217}$, and pTau$_{231}$) present in pooled Subtype 6 CSF were analyzed by Alamar immunoassay following a 24-hour incubation, with increasing concentrations of HSA (by volume: 0.001%, 0.01%, 0.1%, 1%, 2%). HSA addition did not significantly decrease endogenous levels of any phosphorylated tau species. Significance was assessed for the final concentration by 1-way ANOVA; (*p≤0.05; **p≤0.01; ***p≤0.001, ****p≤0.0001).
